# Case of Tumour in the Brain

**Published:** 1846-01

**Authors:** E. Gibson Smith


*Case of Tumour in the Brain.* By E. Gibson Smith, M.D.

Mr. J. B., of good constitution and temperate habits, ætat. 31, sent for me in the afternoon of the 25th of July last, and complained of a severe pain in the back of his head, accompanied with constant vomiting. He begged of me to quiet the irritability of his stomach, as his efforts in vomiting caused him such exquisite pain in his head, that—as he expressed himself—"it would drive him crazy." The pulse was scarcely disturbed.

After examining the evacuation from his stomach, and finding it principally composed of bilious matter, I looked upon it as a case of engorgement of the liver, and prescribed a solution of salt of tartar, to be followed as soon as the stomach became quiet by pills composed of hydrarg. chlor. mit., with pulv. rhei. and ext. colocynth. Towards evening he was much improved. The solution was continued at longer intervals, and he passed a tolerably comfortable night. At daybreak his bowels were profusely evacuated, the dejection being of a very dark colour, and exceedingly offensive.

On the 26th he was much relieved; complained of lassitude, with a very little headache. I determined to keep up an action upon his bowels, and ordered mass. hydrarg. grs. xx., ext. colocynth. ext. rhei, *aa.* grs. xv, to be made into twelve pills; one every two hours; and at the same time applied a blister to the nape of the neck.

He vomited several times in the course of the afternoon, but it occasioned him little or no uneasiness, was not accompanied with nausea, and ceased after taking one or two tablespoonfuls of the solution: *pulse natural.*

27th. Very much better; quite cheerful; complained of weakness, and inclined to remain quietly in bed; bowels opened again, and looked more natural: *pulse natural.*

28th. Was informed that he had passed a comfortable night. Some little headache, but occasioning scarce any inconvenience; discontinued all the medicine.

29th. Met him at his place of business, and with the exception of weakness, felt perfectly well.

On the afternoon of the 30th I was requested to see him again. Upon reaching him, found him with a repetition of all the symptoms observed at the first visit. This time, however, the eyes were much injected, and the light was unpleasant. Pulse slightly excited, about 90, full, but not tense, easily compressed; tongue heavily coated; breath foul; skin warm, but not dry nor burning. Prescribed calomel, 10 grains, powdered jalap, 15 grs. As I had made arrangements to leave the city early the next morning, I requested my friend, Professor Huston, who was visiting a patient in the same hotel, to see him for me the next morning.

I remained away three days, not thinking but my patient would recover as speedily as he had done before. Upon my return, however, Dr. Huston informed me that he had continued vomiting the whole of the 31st, suffered greatly from pain in the occipital region, and that in consequence of his bowels not being moved, he had ordered hydrarg. chlor. mit. ℈j, to be given him in the evening. At his visit in the morning of the 1st of August, found him still without an evacuation; vomiting not so frequent, but still occasional; prescribed ext. sennæ fl. ℥ij. A teaspoonful every two hours.

2d. No evacuation; pain in the head somewhat relieved; vomiting occasional; ordered enemata, which, however, only emptied the rectum.

3d. I took charge of my patient. Pulse natural, rather weak; skin dry, but not hot; tongue somewhat cleaned off; had not vomited all day; abdomen soft; no tympanitis; no pain upon pressure. Ordered hydrarg. chlor. mit. grs. iv, ext. colocynth, grs. xii, pulv. gambogia grs. ij, tart. ant. grs. ss, to be made into eight pills, one every two hours; and a mixture composed of liq. ammon. acet. ℥vj, spts. nit. dulc. ʒvj, sacch. alb. ʒvj, to be taken in desert-spoonful doses, at intervals of two hours.

4th. Rather better, but no evacuation; no headache; can converse, is even cheerful; pulse 80 and natural. After waiting till evening for a motion, and finding the obstruction to continue, I prescribed ol. tiglii in capsules, each containing half a drop. One for a dose, at intervals of two hours.

5th. A profuse evacuation from the bowels this morning; no headache; has not vomited for twenty-four hours; complains of weakness, no inclination to move; appetite improved. Omit all the medicine.

6th. No improvement upon yesterday; a feeling of exhaustion; countenance somewhat anxious; no headache, but listless; does not wish to converse.

7th. His friends thinking that he was not as comfortably situated as he might be, at the hotel, had him removed to a relation's, a mile distant; obliged him to dress himself, although greatly averse to it, and walk down to the carriage from the fourth story, a proceeding which altogether occupied an hour and three quarters. Feeling annoyed at the precipitancy used by his friends, (as it was done entirely without my knowledge,) I begged to withdraw from the management of the case, but was solicited so strongly by the patient to remain, that I at length consented to do so.

Dr. Tucker was called in consultation on the same day. After an examination, we concluded to administer a grain of the sub mur. hydrarg. every hour, until the bowels relaxed or the gums were touched. We agreed perfectly in the opinion that as soon as we could establish a regular action of the bowels, all the other symptoms would disappear.

The patient bore his removal tolerably—at least he did not complain very much after he had got comfortably fixed in bed. Very little headache; skin pleasant; pulse scarcely excited; vomited once or twice within an hour after his removal.

8th. Was informed this morning that he had passed a comfortable night, but that about 7 o'clock, A. M., he had vomited matter so exceedingly offensive, that it was barely possible to remain in the room. His nurse, however, had thrown it away, and question as we would, we could not discover whether it was feculent matter or not. We at once came to the conclusion that there *must* be some unusual impediment in the bowels, and became very anxious for the safety of the patient.

No marked change all day; rather listless; sometimes a question had to be repeated before an answer could be obtained; tongue pretty heavily coated; a little headache; no disposition to eat; pressure upon the left side of the umbilicus occasioned some pain, but nowhere else. Enemata were administered, but came away without bringing any feculent matter.

9th. This morning, found the patient pretty much the same, but as yet no evacuation. Ordered ol. tiglii in half-drop doses, to be given every two hours; fomentation to the abdomen, with occasional draughts of the froth of porter. He was allowed some beef tea and a little wine whey—iced—as the weather was very warm. In the evening, no marked change; pulse still as nearly natural as possible; very slight tympanitis; some eructation, but no vomiting; no pain in the head; skin a little warmer than natural.

Dr. Tucker was detained by some accident from meeting me, and the family proposed to send for a Homœopathic physician. I therefore immediately withdrew; but meeting Dr. Tucker in the morning, begged him to remain, as he was their family physician, and I felt very loth to leave the patient in the hands of the Homœopathist.

On the morning of the 10th a large evacuation from the bowels took place, and accordingly the Homœopath was duly credited, although his prescription consisted of sup. carb. soda ℨj, sacch. lact. ℨj, pulv. digitalis gr. j, to be divided into twelve powders, one to be given every two hours; and yet this had overcome the most obstinate constipation!

From this time—as I learned subsequently—no particular difficulty occurred in procuring proper discharges from the bowels. His condition, however, remained pretty much the same, the symptoms varying but slightly from those which occurred during the first of his illness. A cold infusion of senna, made by placing a half ounce of senna in a pint of cold water, and given in teaspoonful doses, every two hours, would operate very pleasantly in the course of the next twenty-four hours, until within a week of his death, when the pain in his head increased, and the muscles at the back part of his head became paralysed, so that his chin nearly rested on his sternum. Priapism began to occur; his bowels again became confined—resisting entirely the action of medicine—until the morning of the 9th of Sept., when he insisted upon getting up to the stool. His male nurse observing that he was urinating upon the floor, discovered that he had violent priapism, and upon attempting to bend the penis down into the vessel, the patient suddenly gasped and expired.

*Autopsy, twenty-four hours after death.*

Present, Drs. Tucker, Ashmead, Vanstavoren and Duhring.

The body has been kept in ice, and there are no signs of decomposition.

The Brain. The large vessels upon the upper surface of the cerebrum were filled with blood, otherwise it was of a healthy appearance. Upon detaching the brain from the medulla spinalis and turning it over, an indurated tumour was discovered in the left cerebellum. It was separated from the surrounding cerebral substance with the greatest possible ease; was of a scirrhous hardness, its surface being very irregular and knotted. Its length was about 2¾ inches—width 1¾ inches—thickness ¾ inch, and weighed six drachms. It was composed of a number of small tumours, varying from the size of a millet seed to that of a cherry stone. On the surface, these tumours appeared rounded, but in the interior of the mass, when cut, they seemed to be irregular cuboids, of a reddish colour upon their exterior, owing to their being covered with a highly vascular cellular tissue; internally, perfectly white, of a uniform consistence, resembling cartilage, but softer, and all united firmly together by vascular cellular tissue. Its long diameter lay parallel with the horizontal divisions of the cortical substance. The anterior extremity extended to the outer edge of the left crura cerebri—its posterior extremity about one-quarter of an inch from the raphae of inferior surface of cerebellum, and at the distance of 1½ inches from the pons var. it approached within a line of the upper surface of the cerebellum. Here some of the cineritious substance adhered to the superficial edge of the tumour, which on being pressed between the fingers, felt in places quite hard. The whole of the superior surface of the cerebellum was softer and paler than natural, and for half inch in width over the posterior edge of the tumour very pallid, or rather of a pale yellowish red; soft, almost diffluent. The medullary portion of the cerebellum in contact with the tumour, soft, pulpy, of a yellowish tinge, with a very slight shade of red. The cerebellum on the right side perfectly natural. Pons varolii and medull. oblongata, rather soft. The arachnoid and pia mater just around the tumour, were very much injected, but otherwise were perfectly natural. There was about two ounces of clear serum around the medulla oblongata.

Chest. The heart and lungs, viewed superficially, appeared natural.

Abdomen. The stomach, from near its cardiac orifice to the pylorus, empty, and contracted to size of the small intestine; about a gill of fluid in the large extremity; a good deal of mucus on its mucous coat, but of a natural texture generally, except about three-quarters of an inch square of the long extremity, where there was softening of the membrane, with injection of its large vessels, and some extravasation in its tissue. The liver, omentum and bowels appeared perfectly natural.

